# Appraisal of a Simple and Effective RT-qPCR Assay for Evaluating the Reverse Transcriptase Activity in Blood Samples from HIV-1 Patients

**DOI:** 10.3390/pathogens9121047

**Published:** 2020-12-13

**Authors:** Beatrice Macchi, Caterina Frezza, Francesca Marino-Merlo, Antonella Minutolo, Valeria Stefanizzi, Emanuela Balestrieri, Carlotta Cerva, Loredana Sarmati, Massimo Andreoni, Sandro Grelli, Antonio Mastino

**Affiliations:** 1 Department of Chemical Science and Technology, University of Rome “Tor Vergata”, 00133 Rome, Italy; macchi@med.uniroma2.it; 2 Department of Systems Medicine, University of Rome “Tor Vergata”, 00133 Rome, Italy; catafrezza@hotmail.it (C.F.); carlottacerva@gmail.com (C.C.); sarmati@med.uniroma2.it (L.S.); andreoni@med.uniroma2.it (M.A.); 3 Department of Chemical, Biological, Pharmaceutical, and Environmental Sciences, University of Messina, 98166 Messina, Italy; fmarino@unime.it; 4 Department of Experimental Medicine, University of Rome “Tor Vergata”, 00133 Rome, Italy; antonella.minutolo@uniroma2.it (A.M.); valeriastefanizzi1995@gmail.com (V.S.); balestrieri@med.uniroma2.it (E.B.); grelli@med.uniroma2.it (S.G.); 5 Tor Vergata University Hospital, 00133 Rome, Italy; 6 The Institute of Translational Pharmacology, CNR, 00133 Rome, Italy

**Keywords:** human immunodeficiency virus, HIV diagnostics, viral load, reverse transcriptase, quantitative PCR assay

## Abstract

Testing HIV-1 RNA in plasma by PCR is universally accepted as the ultimate standard to confirm diagnosis of HIV-1 infection and to monitor viral load in patients under treatment. However, in some cases, this assay could either underestimate or overestimate the replication capacity of a circulating or latent virus. In the present study, we performed the assessment of evaluating the HIV-1 reverse transcriptase (RT) activity by means of a new assay for the functional screening of the status of HIV-1 patients. To this purpose, we utilized, for the first time on blood samples, an adapted version of a real-time RT quantitative PCR assay, utilized to evaluate the HIV-1-RT inhibitory activity of compounds. The study analyzed blood samples from 28 HIV-1-infected patients, exhibiting a wide range of viremia and immunological values. Results demonstrated that plasma HIV-1 RT levels, expressed as cycle threshold values obtained with the assay under appraisal, were inversely and highly significantly correlated with the plasma HIV-1-RNA levels of the patients. Thus, an HIV-1 RT quantitative PCR assay was created which we describe in this study, and it may be considered as a promising basis for an additional tool capable of furnishing information on the functional virological status of HIV-1-infected patients.

## 1. Introduction

The key aim of the combination anti-HIV-1 antiretroviral therapy (ART) was to block the progression of the disease, significantly reducing virus levels in plasma in most of patients but without leading to the eradication of the virus. As a result, HIV-1 infection has taken on the characteristics of a chronic infection in properly treated patients. However, the risk of exacerbation of the infection is always there. In fact, chronicization of infection in ART patients has encouraged the establishment of “reservoirs”, in which, potentially, a replication-competent virus persists. The nature and clinical significance of these HIV-1 “reservoirs” seem rather heterogeneous and difficult to precisely quantify [1,2]. In fact, evaluation of the plasma viral load cannot give information on the replication-competent reservoir. Moreover, about 5–30% of ART patients fail to significantly improve their number of CD4+ cells even after achieving apparently obvious virological success. On the other hand, discordant HIV-1 patients undergoing ART, who, for years, stably maintained acceptable CD4+ cell levels despite sustained viral loads (VLs) of 500–5000 copies/mL, have been described [3]. A virus from this type of discordant responder was found to have significantly lower viral replication capacities, probably due to the presence of defective viral particles. Thus, the possibility exists that, in some cases, viremia, expressed in terms of VLs, calculated by accounting the number of RNA viral copies, could either underestimate or overestimate the replicative potential of the HIV-1 burden.

A functional, not cell-based, method for measuring the potential replicative capacity of retroviruses was used to evaluate retrotranscriptional activity. Classical methods for evaluating reverse transcriptase (RT) activity were initially based on ascertainment of incorporated radiolabeled nucleotide or digoxigenin-labeled dUTP into acid-insoluble polynucleotides [4,5]. Different strategies were successfully used to improve biosafety while ensuring sensitivity of the assays, including the polymerase chain reaction (PCR)-based methods [6,7,8,9,10,11,12,13,14,15,16,17,18]. We developed two methods for the detection of retrotranscription using conventional PCR and real-time PCR, respectively, to assess cDNA production [19,20]. These assays allowed us to evaluate the HIV-RT inhibitory activity of compounds with a cell/virus free assay, using both commercial HIV-RT and RT from supernatants of HIV-1 chronically infected cell lines as a source of the DNA polymerase enzyme [20]. Moreover, we also developed and used assays based on the same principle to quantify HTLV-1 RT activity in short-term PBMC cell cultures from blood samples of HTLV-1-infected patients [21,22], showing a clinical potential for assays conceived in such a way.

The present study focuses on the need to improve existing assays for the functional screening of the replicative potential of the viral reservoir of patients affected by HIV infection. For this reason, based on the work already realized in the laboratory in which this study was performed, we utilized, for the first time, an adapted version our real-time RT quantitative PCR (RT-qPCR) assay to evaluate HIV RT activity in blood samples from HIV infected patients. This study examined blood samples from HIV-1 infected patients exhibiting a wide range of viremia and immunological values and had the characteristics of a proof of concept study to verify the feasibility of using such an assay.

## 2. Materials and Methods

### 2.1. Patients and Samples

This retrospective cohort study was carried out using plasma sampled from persons infected with HIV-1, showing a viral load (VL) higher than 5 × 10^3^ HIV RNA copies/mL (*n* = 30; VL, range 6523 to 1,075,865 copies/mL) and HIV-1 negative controls (*n* = 15) in 9 mL vacuum tubes containing sodium heparin (NaHeparin) and ethylene diamine tetra-acetic acid (EDTA). Patient blood was sampled from HIV-1-infected adult patients attending the Infectious Disease Center, Policlinico Hospital of Tor Vergata, University of Rome “Tor Vergata”, Rome, before antiviral therapy was initiated or very early since initiation of therapy (immunological characteristics of the patients available in Supplementary Materials, Table S1). All subjects gave their informed consent for inclusion before they participated in the study. The study was conducted in accordance with the Declaration of Helsinki, and the protocol was approved by the Ethics Committee of the University of Rome “Tor Vergata” (trial register number 88/14). Within 6 h after the blood draw, tubes were centrifuged at 700× *g* for 10 min with low brake to separate plasma from cells. The plasma was collected and centrifuged for a further 5 min with full brake to pellet contaminating cells. Plasma was aliquoted and stored at −80 °C until use.

### 2.2. Isolation of HIV RT from Plasma Samples

For detecting HIV-1 RT activity from the virus load in stored plasma, samples were gently thawed on ice and clarified by centrifugation at 10,000× *g* for 5 min. Volumes of 250 µL of plasma were then transferred in new vials and centrifuged at 21,000× *g* for 2 h at 4 °C. Supernatants were removed and virion pellets were lysed by adding 100 µL of lysis buffer (50 mM Tris HCl, 50 mM KCl, 10 mM MgCl_2_, 0.5 mM EGTA, 2 mM DTT, 0.06% NP-40) to each tube and vortexed on ice every 2–3 min for a total of 20 min. Samples containing RT isolated from viral lysates were then stored at −80 °C until their use in the RT activity assay, as described below. Plasma samples from non-HIV-1 positive donors were processed as those from HIV-1 positive patients.

### 2.3. Preparation of RNA Template by glycoprotein D (gD)-Enriched RNA Extraction

The RNA template, enriched for gD specific mRNA, was isolated from glycoprotein D (gD) expressing transfectants, I143tk cells, ectopically expressing the US6 gene of herpes simplex virus 1 [23], using the “Nucleo Spin RNA II“ (Macherey-Nagel, Duren, Germany), according to the manufacturer’s instructions. RNase-free H_2_O (50 μL) supplemented with 20 U RNase inhibitor was added, and then solutions were stored at −80 °C until use. RNA was analyzed using a 1% agarose gel electrophoresis to ascertain the quality of RNA and the absence of DNA contamination and quantified using the Nanodrop ND-1000 (Thermo Fisher Scientific, Waltham, MA, USA).

### 2.4. Primers and Probe for RT Activity Assay

A pair of primers [20] and a probe, specific for the herpes simplex virus 1 (HSV-1) glycoprotein D (gD), were designed based on the nucleotide sequence published in GenBank library (accession no. L09242) using Vector NTI 8 software (Life Technologies, Waltham, MA, USA). The sequence of the forward primer, named gD-Fw, was 5′- CCGGAAACAACCCTACAACC -3′, and the sequence of the reverse primer, named gD-Rev, was 5′- GCATTCGGTGTACTCCATGAC -3. The TaqMan gD-probe sequence was 5′- TCGCTTGGTTTCGGATGGGAGGCA -3′, which was labeled with the fluorescent reporter dye FAM (6-carboxyfluorescein) at the 5′ end and with the TAMRA (6-carboxytetramethylrhodamine) fluorescent quencher at the 3′-end. A 91-bp product was expected when these primers were used. The primers and probe were synthesized and labeled by Metabion International AG, Germany.

### 2.5. RT Activity Assay

For detecting the RT enzymatic activity in blood-derived samples. a method previously described by us, following appropriate modifications, was utilized [20]. Briefly, the RT activity assay was set up in two steps. The first step was a reverse transcription reaction using total gD-enriched RNA as a template and lysates from peripheral blood samples as a RT source. The second step was a TaqMan real-time PCR assay for amplification and quantitative evaluation of specific reverse-transcribed gD-cDNA. The reverse transcription reaction was carried out in a mixture containing 150 ng of RNA, 0.5 μM gD-Rev reverse primer, 0.2 mM dNTP (each), 1× RT buffer (Promega, Madison, WI, USA), and an amount corresponding to 20% of the final volume of the above-described home-made RT preparation from plasma samples. Quantitative real-time PCR amplification was conducted using 1× TaqMan Master Mix, with 0.5 μM of forward and reverse primer, 0.2 μM of hydrolysis probe, and 4 µL of cDNA template in a final reaction volume of 20 μL. Negative controls (no home-made RT source and no template cDNA) were included in all PCR runs. All reactions were performed in two technical replicates and were carried out in a CFX-96 system (Bio-Rad, Hercules, CA, USA). Real-time PCR thermocycling conditions were set at 95 °C for 15 min, followed by 45 cycles at 95 °C for 15 s and 60 °C for 90 s, and fluorescence was detected after every 60 °C extension step.

### 2.6. Statistical Analysis

The correlations between HIV RT viral load and the HIV RNA viral load assays, following logarithmic transformations of the later values, were analyzed using Pearson’s correlation coefficient (*r*). Calculation of Pearson’s *r*, 95% confidence interval, R square and P (two-tailed) values, and curve fittings were performed using the GraphPad Prism v.8.4.3 software (GraphPad Software, Inc., La Jolla, CA, USA).

## 3. Results

The novel RT-qPCR assay for evaluating the RT activity in blood samples from HIV-1-infected individuals was developed by setting up a variant version of the two-step RT-qPCR-based assay, already described by us for evaluating the efficacy of compounds towards HIV-1 reverse transcriptase [20]. The new method was adjusted, aiming to make the less possible changes to the previous technique. In particular, similar to the previously developed assay, the first step consisted of the phase in which HIV-RT generated a cDNA, using, as a specific template for RT, RNA extracted from cells ectopically expressing the gD protein of HSV-1 and using, as a specific primer, a related targeted oligonucleotide. However, different from the previously described assay, where known amounts of commercial HIV-RT were added for cDNA generation, in the novel assay, aliquots of RT preparations from plasma of HIV-1-infected patients were used for the RNA-dependent DNA polymerase reaction. Additionally, in this assay, gene-specific antisense primers (gD-Rev), in place of random primers or oligo-dT, were used for the cDNA reaction to assure a higher fidelity and specificity in the product generation, thus attempting to improve the sensitivity of the assay. Moreover, the same amount of RNA template (150 ng) as in the previous assay was utilized. An aliquot of RT preparation corresponding to 20% of the final volume of the reaction mixture was considered as optimal for cDNA generation based on preliminary experiments, showing that the lower volume of the same RT preparations led to increased cycle threshold (CT) values, while the addition of the higher volume of the same RT preparation did not decrease or even increase CT values. General information on the sensitivity and specificity of the reaction was obtained by setting experimental conditions alternatively in absence of template, primers, and RT preparations. In the absence of primers, the CT was equal to 40, while fluorescence in samples without RNA or without both RT preparations from patients or commercial HIV-RT was undetectable (data not shown).

Regarding the efficiency of the reaction using samples from HIV-1 negative donors, for five of them, it was undetectable (not amplified, NA) in both replicates and for 1 of them, we obtained NA in one of the replicates and a CT value of 45 for the other replicate. A mean of 42.42 ± 3.7 CT was obtained for all detectable values. However, in one case, we obtained a mean CT value of 34.13. In absence of any preventive information on the accuracy of our assay, we then adopted a very prudent approach. For this reason, a value of 32 Ct was arbitrarily considered as the upper, reasonably acceptable limit for samples of HIV-1 positive patients to include in the successive analysis of the results. Two of the 30 participating patients, showing at least one of the duplicate values over this limit, were then considered not evaluable and were excluded from the final analysis of data. Regarding the efficiency of the reverse transcription using the RT preparations from HIV-1 positive patients, we must remember the characteristics of a preliminary proof of concept of this study. Consequently, in absence of information on the sensitivity of our technique to verify the feasibility of the assay, only patients showing clearly detectable levels of VL were selected for the study.

Results are reported in Table 1 and show that RT activity detected in preparations obtained from HIV-1-positive patients corresponded to a number of CT, expressed as a mean CT value, within the range of 27.65–30.70 CT. We then investigated whether CT values, detected by the novel assay, correlated with virological parameters, namely VL, detected in the same plasma samples of the patients from which the RT preparations were obtained.

**Figure 1:**
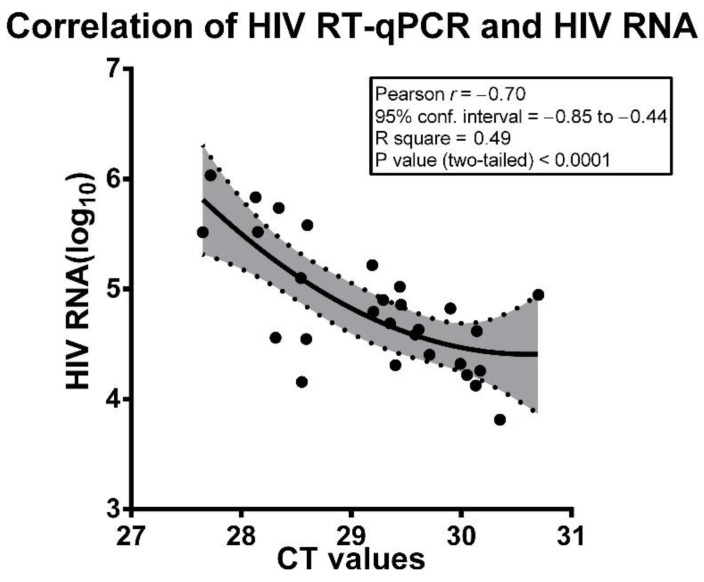
Correlation of HIV load based on HIV RT-qPCR assay and HIV RNA assay (log_10_ values). HIV load based on HIV RT-qPCR assay is expressed as CT values. All data from the 28 analyzed patients are shown as solid points and included in the correlation. The interpolation curve as a solid line and the 95% confidential intervals, delineated by dotted lines and a grey area, are also shown. Statistics are reported in the panel.

Preliminary regression analysis found a nonlinear model of the relationships between the amount of VL and the CT amplification values. For this reason, statistical analysis with Pearson’s correlation coefficient was carried out on VL values expressed as HIV-1 RNA log_10_ copies/mL and crude values of CT obtained with the novel assay. The data presented in Figure 1 allow us to fully realize that CT values obtained with the assay under appraisal were inversely and highly significantly correlated with the level of plasma VL, following log_10_ transformation. This highly significant correlation can be easily appreciated, looking at the fitting curve, including 95% confidence bands, generated using all analyzed data (Figure 1). Analysis of correlations between either the RT assay CT values or log_10_ of VL and immunological parameters of the patients, such as CD4+ and CD8+ cell numbers or CD4+/CD8+ ratios, invariantly gave lower *r* values and lack of statistical significance (Supplementary Materials, Table S2).

## 4. Discussion

Thd discrepancy between the number of RNA viral copies, as determined by commercial real-time PCR-based assays, and real infectious circulating or latent VL was not deeply addressed. In fact, there was no easy solution to this problem. One possible solution is to perform a series of determinations, sufficient to provide statistically correct results, for comparing RNA viral copy values and corresponding infectious values, detected by cell-based assays. In fact, semi-quantitative assessment of infectious virus from patient plasma was carried out in the early era of HIV-1 studies by various methods using donor peripheral blood mononuclear cells (PBMCs) to amplify virus burden before calculation of virus titers usually as the TCID50 per milliliter of plasma [24,25,26,27]. All cell-based methods for infectious virus titration must be carried out in a BSL-3 laboratory. Successive studies on this issue focused their efforts on overcoming two major limits of the above-mentioned methods, i.e., poor efficiency of infection of PBMCs by HIV-1 in vitro and approximate quantization, using different strategies [28]. However, cell-based assays to quantify infectious viral particles, although improved with the ongoing of time, did not produce significant progress in terms of sensitivity, time consumption, and biosafety. Here, we demonstrate that evaluation of RT activity in plasma obtained from blood samples of HIV-1-infected patients, using the method we described, had characteristics that corresponded to an informative and correct functional assay, being highly significantly correlated with plasma VLs of the same patients. However, we cannot exclude the fact that RT activity detected in patient plasma did not necessarily originate only from infectious viral particles but also, at least in part, from genetically defective virions that may have harbored intact RT but were unable to productively infect cells.

However, we must note that, since our study is the feature of a proof of concept study, patients enrolled were purposely selected among those with VL levels > 5 × 10^3^ copies/mL. This choice was made in order to avoid, in this phase of the study, problems of interpretation of results that were biased due to the unpredictable sensitivity level of the assay. Nevertheless, we are conscious that such a threshold is very far from VL levels usually ascertained in patients under therapy for whom such an assay should be intended. In fact, when in some cases, out of the study, we utilized our assay exactly as described here to evaluate RT activity in samples from patients showing VL levels ≤ 5 × 10^3^ copies/mL, results were frequently inconsistent for unknown reasons. This indicates that, while extremely reliable for patients with quite high VL levels, this assay must be properly adjusted to ensure its fully satisfactory application for HIV-1 patients with low VL levels. Studies aimed to ameliorate the sensitivity of our assay, acting on different variables that were actually in progress in our laboratory. For example, the template substrate consisting of total RNA extracted from gD-transfectants could be substituted by a synthetic, gD-specific RNA template generated by in vitro transcription. This device could eliminate the disturbing interference of cellular products other than gD-specific mRNA on the retrotranscriptase activity when very low levels of RT enzyme are present in the reaction mixture.

Moreover, for some individuals, for whom stored, frozen samples of PBMCs were available, we scheduled to detect the RT activity from extracts of these cells. To this purpose, pellets of PBMCs were then gently thawed, washed, and resuspended in lysis buffer before three successive freezing/thawing cycles and preparation of RT extracts, as described for plasma samples. However, CT values obtained using PBMC-derived extracts as a source of RT were always higher from a minimum of one CT to, more frequently, two or three CT values, with respect to those obtained from corresponding plasma samples (data not shown). Thus, these results indicate that levels of RT activity detected in plasma samples were higher than those detected in corresponding PBMC pellets, excluding the fact that the latter procedure could be useful to ameliorate the efficiency of the assay in its present version.

**Figure 2:**
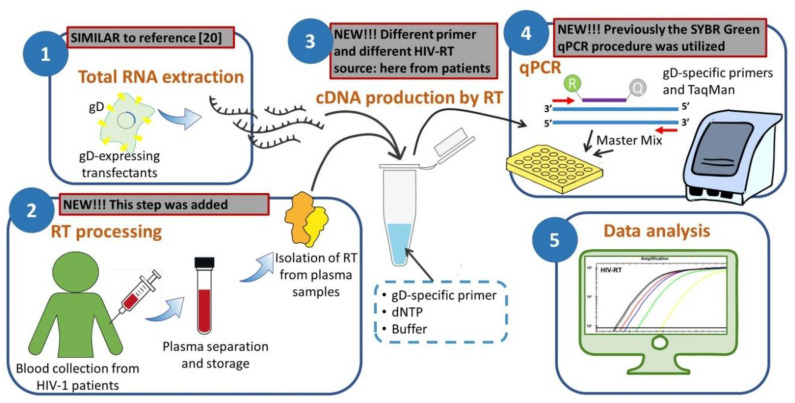
Diagram depicting the assay workflow according to its five main steps. In each step, a note summarizes how it differs from our previous assays.

In conclusion, the findings we report here suggest that the HIV-1 RT assay which we describe for the first time in this study (summarized as a diagram in Figure 2) may be considered a strictly predictive functional assay suitable for evaluating the actual replicative potential of the viral load of HIV-1-infected patients. This conclusion needs to be validated by further studies aimed to assess the capability of the assay to evaluate the RT activity in plasma of patients showing low-level HIV viremia.

## Figures and Tables

**Table 1:** HIV-1 load expressed as copies of HIV-1 RNA, corresponding log_10_, and mean cycle threshold (CT) values obtained with the new reverse transcriptase quantitative PCR assay.

Patient ID	HIV-1 RNA (Copies/mL)	HIV-1 RNA Copies/mL (log_10_)	Mean CT Values
1	6523	3.81	30.35
2	13,304	4.12	30.13
3	14,377	4.16	28.55
4	16,558	4.22	30.05
5	18,053	4.26	30.17
6	20,400	4.31	29.40
7	20,931	4.32	29.99
8	25,300	4.40	29.71
9	35,157	4.55	28.59
10	36,196	4.56	28.31
11	38,664	4.59	29.58
12	41,521	4.62	30.14
13	42,770	4.63	29.61
14	48,786	4.69	29.35
15	62,329	4.79	29.20
16	66,681	4.82	29.90
17	72,111	4.86	29.45
18	79,849	4.90	29.29
19	88,801	4.95	30.70
20	104,832	5.02	29.44
21	126,006	5.10	28.54
22	165,295	5.22	29.19
23	328,191	5.52	27.65
24	330,013	5.52	28.15
25	379,959	5.58	28.60
26	544,912	5.74	28.34
27	683,104	5.83	28.13
28	1,075,865	6.03	27.72

## Supplementary Materials

The following are available online at https://www.mdpi.com/2076-0817/9/12/1047/s1, Table S1: Immunological Characteristics of the Patients; Table S2: Analysis of correlations between either the RT assay CT values or log_10_ of VL with CD4+ T or CD8+ cell count and the CD4+/CD8+ ratio.

## References

[1] Rausch J.W., Le Grice S.F.J. (2020). Characterizing the Latent HIV-1 Reservoir in Patients with Viremia Suppressed on cART: Progress, Challenges, and Opportunities. Curr. HIV Res..

[2] Wang Z., Gurule E.E., Brennan T.P., Gerold J.M., Kwon K.J., Hosmane N.N., Kumar M.R., Beg S.A., Capoferri A.A., Ray S.C. (2018). Expanded cellular clones carrying replication-competent HIV-1 persist, wax, and wane. Proc. Natl. Acad. Sci. USA.

[3] Sufka S.A., Ferrari G., Gryszowka V.E., Wrin T., Fiscus S.A., Tomaras G.D., Staats H.F., Patel D.D., Sempowski G.D., Hellmann N.S. (2003). Prolonged CD4^+^ cell/virus load discordance during treatment with protease inhibitor-based highly active antiretroviral therapy: Immune response and viral control. J. Infect. Dis..

[4] Eberle J., Seibl R. (1992). A new method for measuring reverse transcriptase activity by ELISA. J. Virol. Methods.

[5] Somogyi P.A., Gyuris A., Foldes I. (1990). A solid phase reverse transcriptase micro-assay for the detection of human immunodeficiency virus and other retroviruses in cell culture supernatants. J. Virol. Methods.

[6] Boni J., Pyra H., Schupbach J. (1996). Sensitive detection and quantification of particle-associated reverse transcriptase in plasma of HIV-1-infected individuals by the product-enhanced reverse transcriptase (PERT) assay. J. Med. Virol..

[7] DeStefano J.J., Alves Ferreira-Bravo I. (2018). A highly sensitive aptamer-based HIV reverse transcriptase detection assay. J. Virol. Methods.

[8] Greengrass V.L., Turnbull S.P., Hocking J., Dunne A.L., Tachedjian G., Corrigan G.E., Crowe S.M. (2005). Evaluation of a low cost reverse transcriptase assay for plasma HIV-1 viral load monitoring. Curr. HIV Res..

[9] Heneine W., Yamamoto S., Switzer W.M., Spira T.J., Folks T.M. (1995). Detection of reverse transcriptase by a highly sensitive assay in sera from persons infected with human immunodeficiency virus type 1. J. Infect. Dis..

[10] Lerma J., Palacin J.A. (2000). The expanded suprapubic area as a skin donor site in the treatment of congenital absence of the vagina. Plast. Reconstr. Surg..

[11] Lovatt A., Black J., Galbraith D., Doherty I., Moran M.W., Shepherd A.J., Griffen A., Bailey A., Wilson N., Smith K.T. (1999). High throughput detection of retrovirus-associated reverse transcriptase using an improved fluorescent product enhanced reverse transcriptase assay and its comparison to conventional detection methods. J. Virol. Methods.

[12] Malmsten A., Shao X.W., Aperia K., Corrigan G.E., Sandstrom E., Kallander C.F., Leitner T., Gronowitz J.S. (2003). HIV-1 viral load determination based on reverse transcriptase activity recovered from human plasma. J. Med. Virol..

[13] Malmsten A., Shao X.W., Sjodahl S., Fredriksson E.L., Pettersson I., Leitner T., Kallander C.F., Sandstrom E., Gronowitz J.S. (2005). Improved HIV-1 viral load determination based on reverse transcriptase activity recovered from human plasma. J. Med. Virol..

[14] Pizzato M., Erlwein O., Bonsall D., Kaye S., Muir D., McClure M.O. (2009). A one-step SYBR Green I-based product-enhanced reverse transcriptase assay for the quantitation of retroviruses in cell culture supernatants. J. Virol. Methods.

[15] Pyra H., Boni J., Schupbach J. (1994). Ultrasensitive retrovirus detection by a reverse transcriptase assay based on product enhancement. Proc. Natl. Acad. Sci. USA.

[16] Sears J.F., Khan A.S. (2003). Single-tube fluorescent product-enhanced reverse transcriptase assay with Ampliwax (STF-PERT) for retrovirus quantitation. J. Virol. Methods.

[17] Silver J., Maudru T., Fujita K., Repaske R. (1993). An RT-PCR assay for the enzyme activity of reverse transcriptase capable of detecting single virions. Nucleic Acids Res..

[18] Vermeire J., Naessens E., Vanderstraeten H., Landi A., Iannucci V., Van Nuffel A., Taghon T., Pizzato M., Verhasselt B. (2012). Quantification of reverse transcriptase activity by real-time PCR as a fast and accurate method for titration of HIV, lenti- and retroviral vectors. PLoS ONE.

[19] Frezza C., Balestrieri E., Marino-Merlo F., Mastino A., Macchi B. (2014). A novel, cell-free PCR-based assay for evaluating the inhibitory activity of antiretroviral compounds against HIV reverse transcriptase. J. Med. Virol..

[20] Marino-Merlo F., Frezza C., Papaianni E., Valletta E., Mastino A., Macchi B. (2017). Development and evaluation of a simple and effective RT-qPCR inhibitory assay for detection of the efficacy of compounds towards HIV reverse transcriptase. Appl. Microbiol. Biotechnol..

[21] Macchi B., Balestrieri E., Ascolani A., Hilburn S., Martin F., Mastino A., Taylor G.P. (2011). Susceptibility of primary HTLV-1 isolates from patients with HTLV-1-associated myelopathy to reverse transcriptase inhibitors. Viruses.

[22] Macchi B., Balestrieri E., Frezza C., Grelli S., Valletta E., Marcais A., Marino-Merlo F., Turpin J., Bangham C.R., Hermine O. (2017). Quantification of HTLV-1 reverse transcriptase activity in ATL patients treated with zidovudine and interferon-alpha. Blood Adv..

[23] Medici M.A., Sciortino M.T., Perri D., Amici C., Avitabile E., Ciotti M., Balestrieri E., De Smaele E., Franzoso G., Mastino A. (2003). Protection by herpes simplex virus glycoprotein D against Fas-mediated apoptosis: Role of nuclear factor kappaB. J. Biol. Chem..

[24] Andreoni M., Sarmati L., Parisi S.G., Ercoli L., Rocchi G. (1992). Efficient and reproducible new semimicromethod for the detection and titration of HIV in human plasma. J. Med. Virol..

[25] Coombs R.W., Collier A.C., Allain J.P., Nikora B., Leuther M., Gjerset G.F., Corey L. (1989). Plasma viremia in human immunodeficiency virus infection. N. Engl. J. Med..

[26] Lathey J.L., Fiscus S.A., Rasheed S., Kappes J.C., Griffith B.P., Elbeik T., Spector S.A., Reichelderfer P.S. (1994). Optimization of quantitative culture assay for human immunodeficiency virus from plasma. Plasma Viremia Group Laboratories of the AIDS Clinical Trials Group (National Institute of Allergy and Infectious Diseases). J. Clin. Microbiol..

[27] Rusert P., Fischer M., Joos B., Leemann C., Kuster H., Flepp M., Bonhoeffer S., Gunthard H.F., Trkola A. (2004). Quantification of infectious HIV-1 plasma viral load using a boosted in vitro infection protocol. Virology.

[28] Cornall A., Sharma L., Solomon A., Gorry P.R., Crowe S.M., Cameron P.U., Lewin S.R. (2010). A novel, rapid method to detect infectious HIV-1 from plasma of persons infected with HIV-1. J. Virol. Methods.

