# Supplementary material for: Appraisal of a Simple and Effective RT-qPCR Assay for Evaluating the Reverse Transcriptase Activity in Blood Samples from HIV-1 Patients
Source: Pathogens. 2020 Dec 13;9(12):1047. doi: 10.3390/pathogens9121047 (PMC7763350; doi:10.3390/pathogens9121047)
Supplement: Supplementary file 1 [file pathogens-09-01047-s001.pdf]

Macchi et al. :"Appraisal of a simple and effective RT-qPCR assay for evaluating the reverse transcriptase activity in blood samples from HIV patients"

Supplementary Materials

Table S1 Immunological characteristics of the patients when blood samples for the study were collected.

	HIV-1 RNA (copies/mL)	$CD4^+$ T count (cells/mm^3)	$CD8^+$ T count (cells/mm^3)	CD4+/CD8+ ratio
**1**	6523	57	103	0.55
**2**	13304	293	398	0.74
**3**	14377	530	501	1.06
**4**	16558	407	1312	0.31
**5**	18053	303	944	0.32
**6**	20400	682	1075	0.63
**7**	20931	258	860	0.30
**8**	25300	462	822	0.56
**9**	35157	446	375	1.19
**10**	36196	293	704	0.42
**11**	38664	658	835	0.79
**12**	41521	297	691	0.43
**13**	42770	236	761	0.31
**14**	48786	875	2246	0.39
**15**	62329	82	1100	0.07
**16**	66681	699	698	1.00
**17**	72111	230	2596	0.09
**18**	79849	449	1265	0.36
**19**	88801	279	493	0.57
**20**	104832	1	537	< 0.00
**21**	126006	77	665	0.12
**22**	165295	193	540	0.36
**23**	328191	127	834	0.15
**24**	330013	69	470	0.15
**25**	379959	19	380	0.05
**26**	544912	395	588	0.67
**27**	683104	162	1268	0.13
**28**	1075865	192	237	0.81

Table S2 Analysis of correlations between either the RT assay CT values or log_{10} of VL with CD4+ or CD8+ T cell count and the CD4+/CD8+ ratio.

	CT values vs. CD4+	CT values vs. CD8+	CT values vs. CD4+/CD8+	Vcopy (log_{10}) vs. CD4+	Vcopy (log_{10}) vs. CD8+	Vcopy (log_{10}) vs. CD4+/CD8+
**Pearson r**	0.22	0.10	0.08	-0.35	-0.06	-0.30
**95% confidence Interval**	-0.17 to 0.55	-0.03 to 0.45	-0.31 to 0.44	-0.64 to 0.02	-0.42 to 0.32	-0.60 to 0.09
**R square**	0.05	<0.00	<0.00	0.13	<0.00	0.09
**P value (two-tailed)**	0.262	0.623	0.698	0.064	0.770	0.126
**P value summary**	ns	ns	ns	ns	ns	ns
**N of XY pairs**	28	28	28	28	28	28
